# RETRACTION: Effects of Negative‐Pressure Wound Therapy in the Prevention of Surgical‐Site Wound Infection After Vascular Surgery: A Meta‐Analysis

**DOI:** 10.1111/iwj.70169

**Published:** 2024-12-15

## Abstract

**RETRACTION:** YuX. , XieP. , LiJ.‐Z. , CaoL. , and YeJ. , "Effects of Negative‐Pressure Wound Therapy in the Prevention of Surgical‐Site Wound Infection After Vascular Surgery: A Meta‐Analysis,” International Wound Journal 21, no. 2 (2024): e14695, 10.1111/iwj.14695.PMC1082459837740679

The above article, published online on 31 January 2024, in Wiley Online Library (http://onlinelibrary.wiley.com/), has been retracted by agreement between the journal Editor in Chief, Professor Keith Harding; and John Wiley & Sons, Ltd. Following an investigation by the publisher, all parties have concluded that this article was accepted solely on the basis of a compromised peer review process. The editors have therefore decided to retract the article. The authors did not respond to our notice regarding the retraction.

**RETRACTION:** X. Yu , P. Xie , J.‐Z. Li , L. Cao , and J. Ye , "Effects of Negative‐Pressure Wound Therapy in the Prevention of Surgical‐Site Wound Infection After Vascular Surgery: A Meta‐Analysis,” International Wound Journal 21, no. 2 (2024): e14695, 10.1111/iwj.14695.PMC1082459837740679

The above article, published online on 31 January 2024, in Wiley Online Library (http://onlinelibrary.wiley.com/), has been retracted by agreement between the journal Editor in Chief, Professor Keith Harding; and John Wiley & Sons, Ltd. Following an investigation by the publisher, all parties have concluded that this article was accepted solely on the basis of a compromised peer review process. The editors have therefore decided to retract the article. The authors did not respond to our notice regarding the retraction.

